# Apnea-hypopnea index use among intensive care patients: a case series

**DOI:** 10.1186/1752-1947-8-181

**Published:** 2014-06-06

**Authors:** Bülent Gücyetmez, Hakan Korkut Atalan

**Affiliations:** 1 Intensive Care Unit, International Hospital, Istanbul Cad No: 82 Yesilkoy, 34149 Istanbul, Turkey; 2 Intensive Care Unit, Ataşehir Memorial Hospital, Vedat Gunyol Cad. No:28 Kucukbakkalkoy Atasehir, 34758 Istanbul, Turkey

**Keywords:** Apnea-hypopnea index, AHI, Brain death

## Abstract

**Introduction:**

ApneaLink™ (RESMED-Munich, Germany) is a simple and inexpensive device that determines the apnea-hypopnea index. The sensitivity and specificity of the apnea-hypopnea index are 100 and 87.5%, respectively. Our hypothesis can be used to create a treatment plan using the apnea-hypopnea index for intensive care unit patients.

**Case presentation:**

This treatment plan has been created by determining the apnea-hypopnea index of eight Caucasian patients with a variety of diagnoses. Case 1 is that of a 70-year-old man diagnosed with rectum cancer and scheduled for elective surgery. Case 2 is that of a 65-year-old man diagnosed with rectum cancer and scheduled for elective surgery. Case 3 is that of a 78-year-old woman diagnosed with chronic obstructive pulmonary disease-pneumonia. Case 4 is that of a 26-year-old man diagnosed with head trauma. Case 5 is that of an 80-year-old man diagnosed with cerebrovascular disease. Case 6 is that of a 79-year-old man diagnosed with cerebrovascular disease. Case 7 is that of an 8-year-old girl diagnosed with ventricular septal defect-epidural hemorragia. Case 8 is that of a 42-year-old man diagnosed with subarachnoid hemorrage.

**Conclusions:**

The apnea-hypopnea index can be informative regarding prognosis and outcomes, and helps to take precautions and develop new treatment strategies among critical patients in intensive care. The integration of developments in sleep medicine to intensive care unit practices means that we can be more informed about critical patients.

## Introduction

ApneaLink™ (RESMED-Munich, Germany) is a simple and inexpensive device that determines the apnea-hypopnea index (AHI). The AHI, hypopnea index (HI), and oxygen desaturation index (ODI) are calculated using breath flow, peripheral oxygen saturation, and heart rate (HR) during night’s sleep. The sensitivity and specificity of AHI are 100 and 87.5%, respectively [1]. With these features ApneaLink™ can contribute to the diagnosis and treatment of intensive care patients. Therefore, we present the following eight Caucasian patients from our intensive care unit (ICU) where diagnosis and treatment plan decisions were made using the AHI.

## Case presentation

Case 1 involved a 70-year-old man with a medical history of chronic obstructive pulmonary disease (COPD), hypertension (HT), and rectal cancer. He had a body mass index (BMI) of 28.3kg/m^2^ and a score of 2 on Epworth sleepiness scale (ESS) and was scheduled for an open abdominal surgery. One day prior to the surgery, he performed an apnea test (via ApneaLink™) between 00:00 and 06:00 am which yielded the following results: AHI: 17/hr, AI: 3/hr, HI: 14/hr; ODI: 3/hr, minimum SpO2 (min. SpO2) 79%, and maximum HR (max. HR): 124/min. A decision was made for postoperative ICU follow-up of the patient.

Following the three-hour long surgery, the intubated patient was admitted to the ICU, where he was extubated four hours later and given O_2_ mask support (5L/min). The results of an apnea test conducted on the operation day between 00:00 and 06:00 am were as follows: AHI: 21/hr, AI: 4/hr, HI: 17/hr, ODI: 1/hr, min. SpO_2_: 84%, and max. HR:131/min. It was decided that he should be scheduled for postoperative noninvasive mechanical ventilation (NIMV) support (Table 1).

**Table 1:** Clinical features, apnea-hypopnea index, and decisions taken for the first six cases

	**Age**	**Gender**	**Diagnose**	**BMI (kg/m**^**2**^**)**	**AHI/h**	**AI/h**	**ODI/h**	**SpO**_**2 **_**(%) (min)**	**HR/min (max.)**	**Decision**
**Case 1**	70	Male	Rectum Cancer (open abdominal surgery)	28.3	Preop.; 17	3	3	79	124	Postop. ICU admission
					Postop.; 21	4	1	84	131	Postop. NIMV
**Case 2**	65	Male	Rectum Cancer (Laparoscopic abdominal surgery)	20.3	Preop.; 22	0	3	86	108	Postop. ICU admission
					Postop.; 38	0	1	81	101	Postop. NIMV
**Case 3**	78	Female	COPD	25.2	18	8	5	72	102	Discharge on BIPAP use
**Case 4**	26	Male	Head Trauma (Tracheostomized patient)	19.5	2	2	0	95	109	Discharge on spontaneous respiration
**Case 5**	80	Male	CVD (Tracheostomized patient)	27.7	18	6	3	89	83	Discharge on home ventilator use
**Case 6**	79	Male	CVD (Tracheostomized patient)	20.8	34	3	7	79	97	Discharge on home ventilator use
				**PaCO**_**2 **_**(Pretest)**		**PaCO**_**2 **_**(Pretest)**		**Apne duration**		
**Case 7**	8	Female	Ventricular septal defect (Brain death)	45mmHg		75mmHg		5 minutes		Apnea can be recorded and demostrated
**Case 8**	42	Male	Subarachnoid hemorrhage (Brain death)	41mmHg		76mmHg		9 minutes		Apnea can be recorded and demostrated

AHI, Apnea-Hypopnea Index; AI, Apnea Index; BIPAP, Bilevel Positive Airway Pressure; BMI, Body Mass Index, COPD, Chronic Obstructive Pulmonary Disease; CVD, Cerebrovascular Disease; HI, Hypopnea Index; HR, Heart Rate; NIMV, Non-Invasive Mechanical Ventilation; ODI, Oxygen Desaturation Index; SpO2, Peripheral Oxygen Saturation.

Case 2 involved a 65-year-old man with no known medical history and a diagnosis of rectal cancer. He had a BMI of 20.3kg/m^2^, an ESS score of 2, and was scheduled for a laparoscopic abdominal surgery. One day prior to the surgery, he performed an apnea test via ApneaLink™ between 00:00 and 06:00 am, which yielded the following results: AHI: 22/hr, AI: 0/hr, HI: 22/hr; ODI: 3/hr, min. SpO2 86%, and max. HR: 108/min. A decision was made for postoperative ICU follow-up of the patient.

Following the two-hour long surgery, the intubated patient was admitted to the ICU, where he was extubated at the second hour and given O_2_ mask support (5L/min). The results of the apnea test conducted on the operation day between 00:00 and 06:00 am were as follows: AHI: 38/hr, AI: 0/hr, HI: 38/hr, ODI: 1/hr, min. SpO_2_: 81%, and max. HR: 101/min. It was decided that he should be scheduled for postoperative non-invasive mechanical ventilation (NIMV) support (Table 1).

Case 3 involved a 78-year-old woman with a medical history of COPD, HT, and diabetes mellitus (DM). Her BMI score was 25.2kg/m^2^. She had presented to the emergency department with hypoxia, hypercapnia, tachypnea, and dyspnea, and was admitted to the ICU with a diagnosis of pneumonia. She was followed-up on in the ICU for five days, during the first two of which she was given NIMV support intermittently during the day and continuously at night. She received nasal O_2_ support for the following three days, and on day four she performed an apnea test between 00:00 and 06:00 am under an O_2_ mask (5L/min), which yielded the following results: AHI: 18/hr, AI: 8/hr, HI: 10/hr, ODI: 5/hr, min. SpO_2_ 72%, and max. HR 102/min. It was decided that she should be scheduled to be discharged on bilevel positive airway pressure (BIPAP) support (Table 1).

Case 4 involved a 26-year-old man with no known medical history and a BMI of 19.5kg/m^2^. He was transferred to the ICU unconsciously, with a Glasgow Coma Score (GCS) score of 6 and tracheostomy due to head assault-related trauma. He stayed in the ICU for 26 days and was separated from mechanical ventilation support after 6 days. Twelve days after admission, he performed an apnea test between 00:00 and 06:00 am in the room air, which yielded the following results: AHI: 2/hr, AI: 2/hr, HI: 0/hr, ODI: 0/hr, min. SpO_2_ 95%, and max. HR: 109/min. It was decided he should be scheduled to be discharged as tracheostomized, and with spontaneous respiration under nurse supervision (Table 1).

Case 5 involved an 80-year-old man with a diagnosis of HT and DM. He had a BMI of 27.7kg/m^2^ and presented to the emergency department with a high sleep tendency and plegia on the left side. A right thalamic and cerebellar infarction was detected in his cranial diffusion magnetic resonance imaging (MRI) scan. He was admitted to the ICU with GCS score of 4. He was followed-up on in the ICU for 38 days and tracheostomized on day 21. The results of the apnea test conducted on day 26 between 00:00 and 06:00 am with the tracheostomy mask (5L/min O_2_) were as follows: AHI 18/hr, AI: 6/hr, HI: 12/hr, ODI: 3/hr, min. SpO_2_: 89%, and max HR: 83/min. It was decided he should be scheduled to be discharged as tracheostomized, with home care conditions assured under home ventilator support (Table 1).

Case 6 involved a 79-year-old man with a medical history of myocardial infarction, HT, and cardiac insufficiency. He had a BMI of 20.8 kg/m^2^ and presented to the emergency department with spasms and sudden loss of consciousness. He was admitted to the ICU after a right middle cerebral artery (MCA) infarction was detected in his cranial diffusion MRI. He was followed-up on in the ICU for 16 days and he was tracheostomized on day 11. He performed the apnea test with the tracheostomy mask (5L/min O_2_) on day 15 between 00:00 and 06:00 am. The results were as follows: AHI 34/hr, AI: 3/hr, HI: 31/hr, ODI: 7hr, min. SpO_2_: 79%, and max HR: 97/min. It was decided that he should be scheduled to be discharged as tracheostomized, with home care conditions assured under home ventilator support (Table 1).

Case 7 involved an 8-year-old girl with a diagnosis of ventricular septal defect. She presented to the ICU as intubated after ventricular septal defect (VSD) closure. She was on 7.4mcg/kg/min dopamine, 8mcg/kg/hr dobutamine, and 0.08mcg/kg/min adrenalin support postoperatively. She received a 1mg/hr midazolam infusion for 14 hours and a 0.15mg/kg/hr tramadol infusion for 23 hours. After 24 hours, her pupils were dilated and an epidural hemorrhage and shift were detected in her cranial computational tomography (CT) scan, after which the epidural hemorrhage was extracted. Despite the lack of sedative administration for the first 96 postoperative hours, she was sedated and the conditions of her pupils did not change. A reverse flow was detected in her carotid Doppler scan but no intracerebral hemorrhage was detected in her cerebral angiogram. At 98 postoperative hours, she performed the standard apnea test and her ApneaLink™ recordings were made simultaneously. Due to the desaturation occurring at the end of the apnea test that lasted 4 minutes and 38 seconds, continuous positive airway pressure (CPAP) treatment was applied. Her PaCO_2_ value increased from 45 to 75mmHg and the ApneaLink™ showed that there was no respiratory effort present. This demonstrated that the presence of apnea can be seen and recorded by ApneaLink™ during the apnea test.

Case 8 involved a 42-year-old man with a diagnosis of hypertension who presented to the ICU unconscious and after a 20-minute resuscitation. His pupils were with fixed and dilated, his GCS score was 3, he was orally intubated, and had hypotension following a loss of consciousness and cardiac arrest. After detecting a subarachnoid hemorrhage in his cranial CT scan, he was started on anti-edema therapy and sedative medication. The cerebral angiogram performed twelve hours after admission to the ICU showed that there was no cerebral hemorrhage, and so a standard apnea test was performed with ApneaLink™ recording the apnea data. After nine minutes, his PaCO_2_ value increased from 41 to 76mmhg and ApneaLink™ showed that there was no respiratory effort present. This demonstrated that the presence of apnea can be seen and recorded by ApneaLink™ during the apnea test.

All patients were Caucasian.

## Discussion

The aim of this article is to emphasize and discuss the importance and benefits of determining AHI among ICU patients. Yaggi *et al*. and Meoli *et al*. define apnea as an interruption of nasal airflow for at least 10 seconds, and hypopnea as a decrease in the airflow that is greater than 30% accompanied by a 4% decrease in the O_2_ saturation [2,3]. A polysomnography (PSG) may not always be performed in the ICU but simple, inexpensive, and easy to use devices such as ApneaLink™ can be used to determine AHI [4,5]. The detection of AHI among ICU patients can be beneficial in determining the diagnosis and treatment of the following cases.

### AHI in elective surgery patients

The American Society of Anesthesiologists (ASA) classification [6], age [7], type of muscle relaxant [8], smoking [9], low albumin levels [10], duration of surgery [11], type of anesthesia [6,12], and other comorbidities [9] (like COPD, coronary artery disease, and kidney failure) are associated with the development of complications. Preoperative complications are most frequently observed in abdominal surgeries [13]. In their retrospective study Gupta *et al*. reported that obstructive sleep apnea syndrome (OSAS) patients undergoing orthopedic surgery experience more complications during their postoperative period and have longer hospital stays [14]. Case 1 and Case 2 had high AHI during their preoperative period, independent of their BMIs. In such cases it is likely that the patient may have an obstructive sleep apnea syndrome (OSAS) diagnosis that they are not aware of. Information on preoperative AHI provides an opportunity both to review the anesthesia plan and consider postoperative intensive care follow-up. This feature of AHI may prove that it is useful in practice for the preoperative evaluation of anesthesia. Additionally, both of these cases had an increase in AHI postoperatively, particularly in favor of hypopnea. Postoperative increase in AHI may be associated with the surgical technique used and/or inadequate or excessive analgesia. In such a case, AHI may be used to question the surgical technique and the analgesic regimens. A postoperative AHI increase can lead to atelectasis and may require treatment such as postoperative NIMV support. Under such circumstances, AHI detection in elective surgery patients can provide significant benefits.

### AHI among ICU patients with COPD diagnosis

The comorbidity of COPD and OSAS is defined as an overlap syndrome (OS) [15]. Compared to COPD, more frequent hypoxia, arrhythmia, pulmonary hypertension, and heart failure are observed in OS [16]. OSAS alone has also been shown to increase the risk of stroke and death [2]. Young *et al*. have emphasized that OSAS has a high prevalence and a significant amount of the cases are undiagnosed OSAS [17]. Given this fact, determining AHI among COPD patients followed-up on in the ICU is significant for estimating possible risks and mortality. Case 3 was followed-up on in the ICU with COPD and pneumonia diagnoses. It is unclear whether the patient whose infection status had regressed and clinical condition had enhanced had OSAS in addition to the COPD diagnosis. Researching OSAS by defining the patient’s OS condition would help to understand the possible risks and determine the postoperative intensive care treatment plan more effectively. The patient having high AHI was diagnosed with OS and discharged with BIPAP. Therefore, determining AHI among ICU patients with COPD diagnosis should be standardized during their hospital stay.

### AHI among tracheostomized patients regarding the home ventilator use decision

The incidence rate of tracheostomy among intensive care patients is 24% [18]. A tracheostomy provides advantages such as reducing dead space, increasing patient comfort, reducing airway resistance, and ease of aspiration compared to endotracheal intubation [19]. Hsu *et al*. reported that a tracheostomy applied before 21 days after an unsuccessful weaning reduces mortality, and emphasized that long intubation periods prior to a tracheostomy prolong the ICU stay and negatively affect the success of weaning [20]. In this study, it has been reported that ventilator need increased among tracheostomized patients who developed pneumonia, had a PaO_2_/FiO_2_ value below 250, or were tracheostomized after 21 days. There are objective criteria in the literature on tracheostomy indication, timing, and decannulation indications [20-23]. However, we have more subjective knowledge on the timing and duration of post-tracheostomy mechanical ventilation support. The use of mechanical ventilation support when unnecessary or lack of its use when necessary are both harmful for a tracheostomized patient. Apnea detected in a tracheostomized patient may only be central apnea, and hypopnea presence means hypoventilation and atelectasis, both of which require mechanical ventilation support. Trigeminocardiac reflex-related arrhythmia can be seen in patients who have undergone aneurysm surgery, while apnea can occur in patients diagnosed with intracerebral bleeding [24,25]. Normal AHI values on the other hand show that no mechanical ventilation support is needed. Cases 4, 5, and 6 were tracheostomized for different reasons and the decision whether to use a home ventilator was given based on their AHI. Therefore, AHI can be used as an objective criterion in determining the mechanical ventilation need for tracheostomized patients.

### Detection and recording of apnea during the apnea test in cases of brain death

In case of brain death, an apnea test is an important reliable method to evaluate the medullary respiratory center’s functions [26,27]. Evaluation of the cerebral blood flow is recommended when the results of the ‘apnea test’ are suspected. Prior to the apnea test, it is made sure the patient is normotensive, normothermic, and normocapnic (PaCO_2_ 35mmHg to 45mmHg), with SpO_2_ less than 95% via mechanical ventilation support with FiO_2_ at 100% and positive end expiratory pressure (PEEP) at 5cmH_2_O [28]. An apnea test result is considered as positive if the PaCO_2_ value increases beyond 60mmHg or more than 20mmHg when the patient is separated from mechanical ventilation for 10 minutes and is given 100% oxygen at 6L/minute rate [28]. Complications such as hypoxia, arrhythmia, acidosis, and hypotension associated with being separated from mechanical ventilation can occur during the apnea test [29-31]. In case of a complication, the apnea test is performed with a T-piece or under continuous positive air pressure (CPAP) [32]. A case of cardiac arrest is recorded by showing lack of rhythm via electrocardiography (ECG). Therefore, we believe that recording apnea test positivity along with apnea in the case of a significant clinical condition such as brain death will provide significant advantages. The presence of apnea was recorded via simultaneous ApneaLink™ while a standard apnea test was performed on the patients in Case 7 and 8. While a recorded apnea in brain death provides more objective and significant data, it can also prevent possible complications by shortening the time waiting for apnea.

## Conclusions

AHI can be informative regarding prognosis and outcomes and helps to take precautions and develop new treatment strategies among critical patients in the ICU. The integration of developments in sleep medicine to ICU practices means that we can be more informed about critical patients.

## Consent

For Cases 1 to 6, written informed consent was obtained from the patients for publication of these case reports and any accompanying figures. A copy of the written consent is available for review by the Editor-in-Chief of this journal.

For the minor patient (Case 7), written informed consent was obtained from the patient’s legal guardian(s) for publication of this case report and any accompanying images. A copy of the written consent is available for review by the Editor-in-Chief of this journal.

For Case 8, written informed consent was obtained from the patient’s next of kin for publication of this case report and any accompanying images. A copy of the written consent is available for review by the Editor-in-Chief of this journal.

## Abbreviations

AI: Apnea Index; AHI: Apnea-hypopnea index; ASA: The American Society of Anesthesiologists; BIPAP: Bilevel Positive Airway Pressure; BMI: Body Mass Index; COPD: Chronic Obstructive Pulmonary Disease; CVD: Cerebrovascular Disease; CT: Computational Tomography; CPAP: Continuous Positive Airway Pressure; DM: Diabetes Mellitus; ESS: Epworth Sleepiness Scale; GCS: Glasgow Coma Score; HI: Hypopnea Index; HR: Heart Rate; HT: Hypertension; ICU: Intensive Care Unit; MRI: Magnetic Resonance Imaging; MCA: Middle Cerebral Artery; NIMV: Non-Invasive Mechanical Ventilation; ODI: Oxygen Desaturation Index; OSAS: Obstructive Sleep Apnea Syndrome; OS: Overlap Syndrome; SpO2: Peripheral Oxygen Saturation; PSG: Polysomnography; PEEP: Positive End Expiratory Pressure.

## Competing interests

The authors declare that they have no competing interests.

## Authors’ contributions

BG was involved in the initial writing of the manuscript. HKA provided intellectual contributions to the content of the manuscript as well as editorial assistance. All authors have read and approved the final version of the manuscript.
